# Leveraging the dynamic adaptation process to address LGBTQ+ health equity in New Mexico high schools

**DOI:** 10.3389/frhs.2025.1499508

**Published:** 2025-06-03

**Authors:** Cathleen Elizabeth Willging, Daniel Shattuck, Mary M. Ramos, Bonnie O. Richard, Adrien Lawyer, Elizabeth Dickson, Gregory A. Aarons

**Affiliations:** ^1^ Southwest Center, Pacific Institute for Research and Evaluation, Albuquerque, NM, United States; ^2^ Department of Anthropology, University of New Mexico, Albuquerque, NM, United States; ^3^ College of Population Health, University of New Mexico, Albuquerque, NM, United States; ^4^ Louisville Center, Pacific Institute for Research and Evaluation, Louisville, KY, United States; ^5^ Transgender Resource Center of New Mexico, Albuquerque, NM, United States; ^6^ Department of Psychiatry, ACTRI Dissemination and Implementation Science Center, and Child and Adolescent Services Research Center, University of California, San Diego, La Jolla, CA, United States

**Keywords:** adoption, dynamic adaptation process, health disparities, implementation strategy, LGBTQ+, mixed- methods, schools

## Abstract

**Background:**

Reducing adolescent suicide in the United States is a public health priority, and lesbian, gay, bisexual, transgender, and queer (LGBTQ+) youth are at elevated risk. The Centers for Disease Control and Prevention has identified six evidence-informed school-based practices (EIPs) that enhance health equity and potentially reduce suicide-related behavior for LGBTQ+ students. Guided by the Exploration, Preparation, Implementation, Sustainment (EPIS) framework, we conducted a five-year, community-engaged cluster randomized controlled trial in 42 New Mexican high schools to study the implementation of these six EIPs. This paper assesses the effectiveness, utility, and benefits of the study's implementation strategy—the Dynamic Adaptation Process (DAP), a participatory and multifaceted implementation approach.

**Methods:**

Our convergent parallel mixed-method analysis focused on 22 New Mexico high schools randomized into an implementation condition. Data sources included annual structured assessments of EIP implementation, individual and small-group qualitative interviews with school professionals, periodic debriefs and interviews with implementation coaches, and coach activity logs. We analyzed quantitative data using linear regressions and qualitative data using deductive coding techniques, integrating the results through a joint display.

**Results:**

The schools experienced statistically significant changes compared to their baseline in adopting safe spaces, prohibitions on bullying and harassment based on LGBTQ+ identity, inclusive health education materials, staff professional development, and facilitation of students' access to LGBTQ+ affirming healthcare. We attribute these changes to the impact of the DAP. The DAP facilitated collaboration among school professionals and community organizations to shift knowledge and attitudes and execute contextually responsive implementation strategies. It also fostered relationship-building and leadership, encouraging school leaders to legitimate implementation efforts and champion health equity for LGBTQ+ students.

**Discussion:**

Participatory implementation science models like the DAP can help prioritize health equity for marginalized populations by enabling the uptake of practices likely to contribute to well-being. This mixed-methods study provides a rich example for future research tackling health disparities for LGBTQ+ people in schools and other complex systems.

## Introduction

Since 2010, public health, adolescent health, and school health authorities in the United States have called attention to evidence-informed practices (EIPs) schools can implement to support students who are lesbian, gay, bisexual, transgender, queer and questioning or of other diverse genders and sexualities (LGBTQ+) (1–7). Mounting evidence supports expert recommendations regarding the positive impacts of the EIPs for LGBTQ+ youth (2, 3). Such EIPs include enabling access to safe spaces like Genders and Sexualities Alliances (GSAs) or Safe Zones, having strong bullying and harassment policies in place, and facilitating student linkages to inclusive and affirmative health information and services (4). Given the major physical and behavioral health disparities affecting LGBTQ+ youth nationwide, including elevated rates of suicidal behavior, applying these EIPs in schools could have a substantial public health impact (2). Unfortunately, efforts to implement and scale out EIPs to make schools safer and more supportive of LGBTQ+ youth have lagged nationwide (5, 6).

Initiatives to reduce suicide or improve the well-being of LGBTQ+ students may not reach their full potential due to implementation challenges in schools (7–9). School culture and climate can influence staff willingness to adopt new practices, as can job tenure, level of professional development, and the availability of peer support and resources for implementation (7, 10–12). Organizational and implementation leadership are also essential to advancing new practices in educational and human service settings (11–15). However, leaders require the capacity to be effective champions who can motivate staff and shape their attitudes toward adopting new practices (10, 12). Frameworks and methods from implementation science may help overcome such challenges in school contexts (10, 16–18).

In 2016, we began a five-year cluster randomized controlled trial called “Implementing School Nursing Strategies to Reduce LGBTQ+ Adolescent Suicide” (RLAS) to tackle research-to-practice gaps for decreasing suicidality and other adverse health outcomes among LGBTQ+ students (19). This mixed-method study centered on implementing six EIPs suggested by the Centers for Disease Control and Prevention in New Mexico high schools: (1) identifying safe spaces on campus; (2) prohibiting harassment and bullying based on sexual orientation and gender expression; (3) providing LGBTQ+ inclusive health education curricula; (4) encouraging professional development on safe and supportive school environments; (5) facilitating access to behavioral health providers experienced in caring for LGBTQ+ youth; and (6) facilitating access to medical providers experienced in caring for LGBTQ+ youth (4). Most states have increased the uptake of these EIPs in public schools over the last decade; yet only 15% of secondary schools implement all six despite their association with lower suicide-related behavior (2, 20–25).

The RLAS study stands in contrast to other school-based suicide prevention interventions. A recent systematic review of suicide prevention in high school and university settings examined over forty interventions with before-and-after outcome measures (26). Most interventions were short-term, aimed at individual staff outcomes, and carried out on single campuses. None sought to make systematic or structural changes to sustain interventions or focused on LGBTQ+ students. These results highlight the importance of deeply understanding implementation contexts, employing locally applicable implementation strategies, and concentrating on large-scale change efforts rather than individual-level intervention.

Our study used the Dynamic Adaptation Process (DAP) (27)—an iterative, data-informed, and contextually responsive methodology from implementation science—to train and support Implementation Resource Teams (IRTs) of school community members to usher in EIPs. The DAP provides a four-phase roadmap informed by the Exploration, Preparation, Implementation, Sustainment (EPIS) framework that attends to factors categorized as occurring in the “inner context” (e.g., school staff and students) and “outer context” (e.g., the community in which a school is located and encompassing policy and resource environments) (9, 10, 18). The phases move IRTs through the process of considering new approaches to implementing EIPs via assessment (Exploration); planning to apply EIPs (Preparation); ongoing planning, training, coaching, and actual use of EIPs with *ad hoc* adaptations (Implementation); and maintaining EIPs over time (Sustainment) (27). The implementation period was structured to allow IRTs to focus on the uptake or improvement of two EIPs annually over three years.

Several studies showcase DAP. Its developers successfully applied this process in four county-wide implementations of a child welfare home visitation intervention over three years, allowing for adaptation to local contexts and improvements annually based on prior experience and ongoing planning (27). Evidence is growing for DAP's utility as a strategy for quality improvement (28), assessing barriers and facilitators (29, 30), adapting interventions (31, 32), and examining the integration of new care processes (33, 34). Several international trials employ DAP to optimize treatment and outcomes for a variety of health and mental health conditions (35–39).

We chose DAP because it is an intentionally collaborative approach to adaptation, recognizing adaptation as a necessary part of implementation to integrate into overall planning processes (27, 40). Alongside adaptation, engaging multiple partners and building capacity at several levels for implementation are critical for ensuring the fit of EIPs to local contexts and their eventual sustainment (41). Especially for practices concerning stigmatized populations (e.g., LGBTQ+ people) and topics (e.g., sexual and reproductive healthcare) for which a multitude of factors can complicate “fit” (e.g., socially conservative ideologies) (9, 42), we hypothesized that a participatory approach like DAP would be ideal.

**Figure 1:**
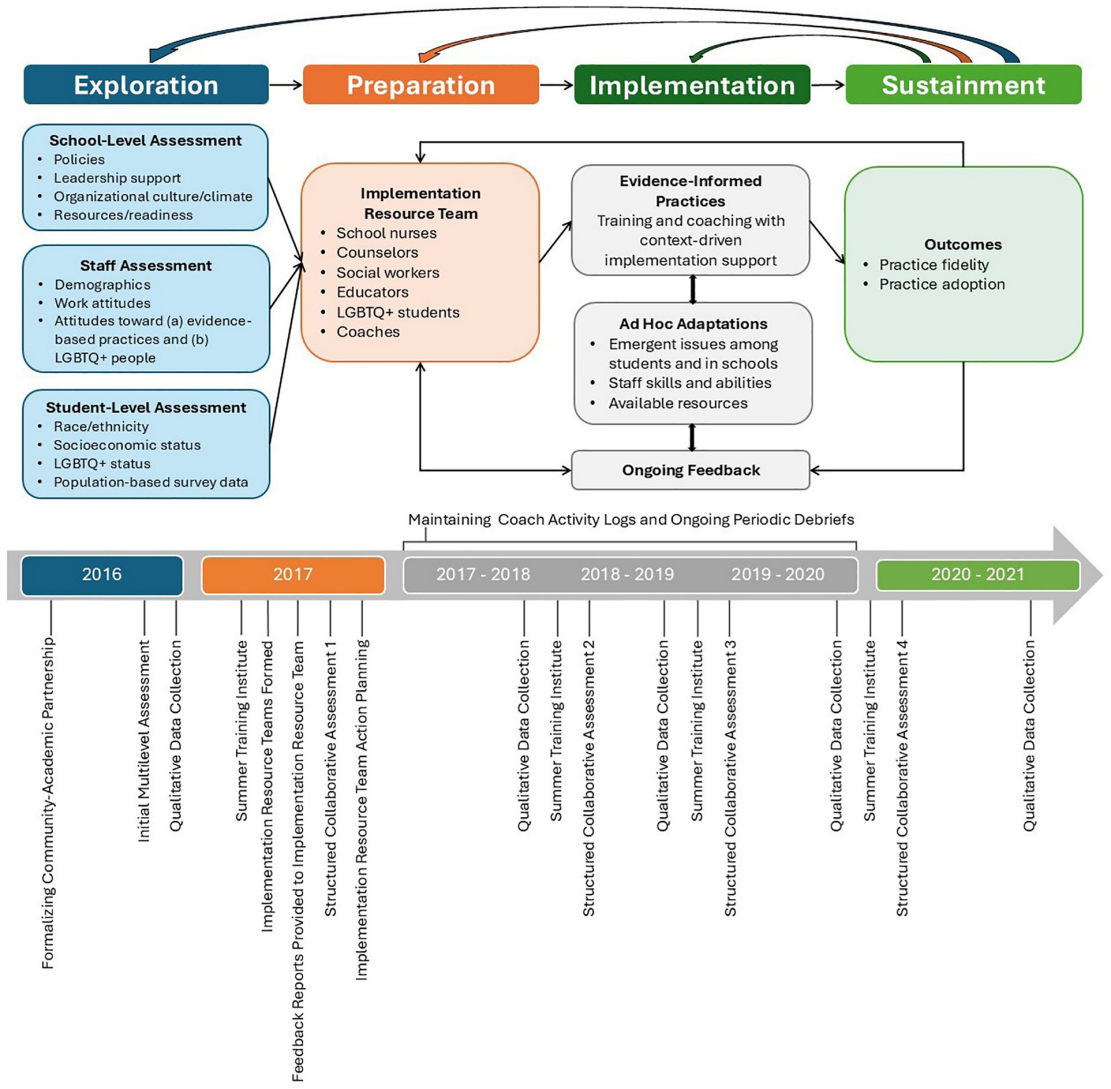
The RLAS dynamic adaptation process.

Per Figure 1, DAP activities in the Exploration Phase included multilevel assessments of school systems, staff, and student data to identify school needs, strengths, and barriers to implementing EIPs. Researchers conducted the assessments and provided feedback to IRTs in the Preparation Phase. In this phase, IRTs participated in initial training to foster consideration of why/what to adapt, what not to adapt, when to seek advice on adaptation, and how to use IRTs for implementation support. Informed by this training and assessment data, and in consultation with an implementation coach, IRTs (a) determined necessary adaptations to school contexts and practices to ensure uptake, (b) decided how to accomplish such adaptations, and (c) created action plans to increase EIP adoption. Training with adaptation support (e.g., coaching) continued into the Implementation Phase when IRTs enacted their plans. Augmentation occurred by adding materials and training in response to school-level barriers (e.g., limited discrimination policies). In the Sustainment Phase, IRTs analyzed successes and challenges related to EIP adoption. As shown in Figure 1, DAP is iterative; IRTs can adjust action plans, create new objectives, and tailor approaches based on short-term impacts to improve long-term outcomes.

A handful of studies describe using DAP across EPIS phases (27, 28, 32, 43). Most published examples are protocol papers (19, 33, 37–39, 44) or about pre-implementation stages and intervention adaptation (31, 36). Most studies concern a single evidence-based clinical practice in child welfare and healthcare settings, and do not discuss IRT or coaching dynamics (28, 32, 43). Critics fault prior DAP studies for lacking “conceptual depth” into how diverse partners may be engaged across implementation processes (41). Our study is the first to use DAP to implement a suite of EIPs supporting the well-being of a socially stigmatized population in schools.

For this analysis, we aim to answer two research questions. First, how successful were IRTs in achieving adoption outcomes? Second, how did the DAP enable implementation of the six EIPs to address health disparities for LGBTQ+ youth? In answering these questions, we describe pivotal issues arising during the four phases of DAP and lessons learned. Our findings complement those from other DAP studies while offering a deeper conceptual understanding of how partners can be fruitfully engaged across EPIS phases (41).

## Materials and methods

### Study context

Our study took place in high schools in New Mexico, a culturally and geographically diverse state ranking 50th in education, 48th in economic well-being, and 50th in overall child well-being (45). The state education department oversees schools organized into 89 districts governed by local school boards. Schools have some flexibility over school management and curriculum implementation. Despite allocating almost half its annual budget to public education (Kindergarten-12th grade), the state has historically struggled to provide sufficient resources, especially for students from low-income families and communities of color (46, 47).

New Mexico has a large population of LGBTQ+ people and progressive policies, with approximately 4.5% of adults (48) and 17.7% of high school students (49) identifying as sexual minorities, and 0.67% of adults (50) and 7.6% of high school students (49) as gender minorities. State law defines LGBTQ+ people as a protected class of citizens and bans conversion therapy for LGBTQ+ youth and insurance exclusions for transgender people (51, 52).

This study emerged from conversations in 2012 between school nurses and a state health officer about the impact of school climates on LGBTQ+ student health. The conversations expanded to include a wider range of school professionals, LGBTQ+ health advocates, and researchers with implementation science backgrounds, culminating in a grant application proposing the DAP for EIP adoption. We formalized our study's 12-member Community-Academic Partnership (CAP) with grant funding in 2016. The CAP helped craft study instrumentation to ensure appropriateness, acceptability, and feasibility for school personnel, created and disseminated implementation resources, and contributed to data analysis.

### School sample

As described elsewhere (53), we recruited 42 high schools randomized into an implementation condition (*n* = 22) or a delayed implementation condition (*n* = 20). Ten implementation schools were in rural areas with a population of less than 2,500. Twelve implementation schools were in urban clusters with a population exceeding 2,500 (54). School size ranged from 25–2,500 students, with a median size of 806. Three implementation schools withdrew during the three-year Implementation Phase due to instability in staffing or changes in school administrative leadership. Upon conclusion of the Implementation Phase, schools in the delayed implementation condition participated in the DAP with coaching support.

### Convergent parallel mixed-method design

We collected quantitative and qualitative data simultaneously and annually from implementation schools during all four EPIS phases (55). All participants contributing data were aged 18 or over and provided written informed consent based on the study protocol approved by the Pacific Institute for Research and Evaluation Institutional Review Board. Figure 1 illustrates the timing of data collection activities and major DAP functions referenced in this paper.

#### Quantitative data collection

At the end of the Exploration Phase and the start of each subsequent school year during the Implementation Phase, IRT members and coaches completed a structured collaborative assessment that established the baseline EIP implementation and allowed us to track adoption. In this way, the assessment served as a data collection tool for the research team and a critical part of the DAP for IRTs to monitor progress and fidelity. Schools in the delayed implementation condition were not asked to complete the structured collaborative assessment to avoid the risk of potential contamination. Thus, we cannot track changes over time for those sites.

The structured collaborative assessment comprised six sections, one for each focal EIP (see Additional File 1). Each EIP had 15–25 practice elements that participants and coaches rated as either “present,” “absent,” or “unknown,” for a total of 49 elements. Unknown elements prompted further research, with IRT members contacting other school staff to find answers. The assessment was completed jointly to capitalize on the combined knowledge of IRT members and facilitate conversations with coaches to resolve discrepancies in opinions or encourage further thinking. These conversations about practices and elements were captured in coaches' activity logs and periodic debriefs for inclusion in the qualitative dataset (see below).

#### Quantitative analysis

We determined the percentage of practice elements implemented to arrive at a current adoption rate for each EIP and overall EIP adoption at each time point. Elements indicated as present were given a score of 1; those absent or unknown were scored 0. Items were summed and then divided by the total number of elements in the assessment. To be in the analysis, the schools completed their structured collaborative assessments for at least two time points. Three had three assessments, one had four assessments, and the majority (15) had five assessments for a total of 19 sites included. Using SPSS (56), we conducted unadjusted linear regression analyses to assess whether adoption rates significantly changed over time for each EIP and the entire suite for the implementation schools. A change was considered significant if the *p*-value was less than 0.05.

#### Qualitative data collection

Qualitative data included individual interviews with school administrators (*n* = 43) and IRT leads (*n* = 50). We also conducted small group interviews with IRT members (*n* = 108). Administrators were principals, assistant principals, and deans of students who consented to their school's participation in RLAS. Both IRT leads and members represented a range of school professionals and sometimes included students and other community members. The interviews were conducted annually by trained researchers with advanced degrees in anthropology or public health at baseline, throughout the Implementation Phase, and in a 1-year Sustainment Phase when coaching and other implementation support were withdrawn, totaling 215 individual and 66 small-group interviews. The first three rounds of interviews took place at school locations, shifting to a virtual format in later years in response to COVID-19 public health measures. We also conducted six individual and one small group interviews with coaches (*n* = 6) during the Implementation Phase at our research office. The digitally recorded and transcribed 45–60 min individual interviews and 60–90 min small group interviews followed semi-structured guides featuring open-ended questions concerning: (1) school and community safety and supportiveness for LGBTQ+ youth; (2) school environmental factors affecting LGBTQ+ students (e.g., policies and procedures, staff training); (3) facilitators and barriers to implementing EIPs; and (4) experiences with implementation efforts, including IRT involvement and coaching. Small group interviews followed traditional protocols for conducting focus groups, and are referred to as such given the size of most IRTs, i.e., fewer than five participants with specialized knowledge and experiences to discuss collectively (57–59). Except for the coaches, participants received a $25 incentive for completing an interview.

Data also included coaches' activity logs and periodic debriefs. The written logs recorded coaches' ongoing engagements with assigned sites, documenting information on interaction type, the people involved, and what transpired. Debriefing with coaches occurred biweekly with one researcher present and again biweekly with the full research team, entailing critical reflections on their work and the changes, challenges, and successes observed in schools (60). Staff recorded written notes to capture coaches' reflections and dialogue on implementation progress.

#### Qualitative analysis

We deductively coded interview transcripts, coaching logs, and periodic debriefs to identify key issues surrounding the use of DAP for EIP implementation across EPIS phases (57). Examples of codes related to EIPs included “suicide prevention,” “LGBTQ+ competency,” “school safety,” “bullying policies,” “health education,” and “referral.” Examples concerning DAP components included “coaching,” “assessment,” and “adaptation.” We also sought to identify barriers (e.g., “staff turnover”) and facilitators (e.g., “supportive administrators”) to EIP usage emerging at baseline and over time.

We assembled “school reports” or detailed case summaries consolidating key information from the transcripts, log entries, and debriefing notes per site that could be easily managed and searched using Dedoose software to assess what happened during each implementation year (61). Reports were organized under various domains, including the EPIS phase, DAP activities, and specific EIPs. Next, we employed a cross-case pattern analysis approach, wherein we compared and contrasted report sections to identify and explore patterns within given domains (57). For example, summaries of experiences for establishing an IRT were compared to identify cross-site commonalities related to that activity. Example patterns related to these experiences included (1) the importance of a diverse team, (2) the need to rely on individuals other than school health professionals to lead the team, and (3) the value of including team members identifying as LGBTQ+ persons. By leveraging our multiple data sources and the rich description in the school reports, this approach enabled a deep, yet generalizable, understanding of patterns related to experiences, activities, and other factors influencing the use of DAP to implement EIPs.

We presented summaries of the preliminary findings to our CAP during quarterly meetings. CAP meetings served as ongoing consensus gatherings where findings from various data sources were discussed and refined through conversations between researchers and community experts (62, 63). As we entered the Sustainment Phase, researchers, coaches, and a subset of CAP members met to distill further and organize qualitative findings.

#### Joint display integration

To assist interpretation of results in consultation with our CAB, we integrated quantitative and qualitative findings regarding EIP adoption using principles of joint display analysis, juxtaposing findings from both datasets within related domains (64, 65). For example, results from quantitative adoption measures of a specific EIP were displayed alongside qualitative findings that helped explain the significance of barriers, facilitators, successes, and challenges pertinent to that same EIP and its adoption.

## Results

In the following sections, we share study findings on EIP adoption and using the DAP to enable uptake. First, we describe the sample of participants from the implementation condition schools. Second, we review quantitative results for EIP adoption. Third, we present qualitative results on DAP functioning to contextualize and explain how EIP adoption outcomes were attained. This presentation summarizes qualitative findings from all data sources to construct a coherent narrative that offers insight into DAP functioning by EPIS phase, quoting participants to illustrate findings. Given the previous “black box” treatment in scientific writing about the DAP (66), we embraced a more descriptive approach for this presentation to yield a grounded understanding of the model's inner workings when operationalized. Fourth, we put forth a joint display demonstrating the intersection with qualitative findings pertinent to EIP adoption.

### School staff participant demographics

Per Table 1, 201 unique individuals completed at least one data collection activity. Participants could select all applicable options when reporting gender identity, sexual orientation, and race. Most identified as heterosexual, and there were large percentages of racial and ethnic minorities. Participation varied yearly due to turnover naturally occurring in schools.

**Table 1:** Implementation condition participant demographics.

Distribution	Y1%	Y2%	Y3%	Y4%	Y5%	Unique^a^
Number of participants	69	98	108	86	68	201
Race (select all that apply)						
American Indian, Alaska Native, Indigenous Latin American	8.7	10.2	8.3	5.8	7.4	10.0
African American, African Descendent, or Black	5.8	6.1	2.8	3.5	4.4	5.0
Middle Eastern American or Middle Eastern	1.4	1.0	0.0	0.0	1.5	1.0
Asian American or Asian	0.0	1.0	0.9	0.0	1.5	1.0
European American, White, Anglo, or Caucasian	73.9	58.2	71.3	67.4	72.1	72.1
Different race	18.8	13.3	16.7	3.5	4.4	13.9
Prefer not to answer	0.0	0.0	0.0	4.7	8.8	0.5
Missing	0.0	18.4	2.8	17.4	1.5	0.0
Ethnicity						
Hispanic	39.1	30.6	38.0	32.6	38.2	42.8
Not Hispanic	60.9	52.0	58.3	51.2	58.8	56.2
Prefer not to answer	0.0	0.0	0.0	0.0	1.5	0.0
Missing	0.0	17.3	3.7	16.3	1.5	1.0
Gender identity (select all that apply)						
Female	72.5	63.3	68.5	62.8	75.0	70.1
Male	24.6	15.3	27.8	20.9	22.1	27.4
Transgender Man/Transman	0.0	1.0	0.9	0.0	0.0	1.0
Transgender Woman/Transwoman	1.4	1.0	0.0	0.0	0.0	0.5
Genderqueer/Gender Nonconforming	1.4	1.0	2.8	1.2	1.5	1.0
Different identity	1.4	0.0	0.9	0.0	0.0	0.5
Prefer not to answer	0.0	1.0	0.0	0.0	0.0	0.5
Missing	0.0	17.3	1.9	15.1	1.5	0.0
Sexual orientation (select all that apply)						
Bisexual	5.8	4.1	8.3	5.8	5.9	6.0
Heterosexual	87.0	65.3	75.0	61.6	80.9	79.6
Lesbian or Gay	7.2	8.2	11.1	10.5	11.8	10.4
Queer	0.0	2.0	2.8	1.2	1.5	2.0
Questioning	0.0	1.0	0.9	0.0	0.0	0.5
Different orientation	2.9	0.0	1.9	1.2	0.0	1.5
Prefer not to answer	0.0	4.1	2.8	5.8	1.5	3.0
Missing	0.0	17.3	1.9	15.1	1.5	0.0

^a^ Calculated based on all unique individuals involved with the study, unweighted by years of involvement.

### Quantitative results: adoption of EIPs

Implementation schools, on average, had approximately 43% of the practice elements outlined in the initial structured collaborative assessment. By the Implementation Phase's close, the schools, on average, implemented roughly 80% of the practice elements in the assessment. We used simple linear regression to test whether these changes in adoption over time were significant. Table 2 shows that the overall regression was statistically significant (*p* < .0001), meaning schools using DAP made substantial strides in adopting the suite of all six EIPs.

**Table 2:** Linear regression results testing changes in evidence-informed practice adoption.

Practice	Linear Regression Results	
Practice 1: Prohibit harassment and bullying based on a student's perceived or actual sexual orientation or gender expression.	R Square	.205
	B (SE)	.082 (.017)
	95% CI	.047–.117
	Sig.	*p* < .0001*
Practice 2: Provide “safe spaces” such as the school health office, counselor's office, designated classroom, or student organization where LGBTQ+ youth can receive support from administrators, teachers, other school staff, or other students.	R Square	.246
	B (SE)	.079 (.015)
	95% CI	.049–.108
	Sig.	*p* < .0001*
Practice 3: Provide health education curriculum or supplemental materials that include HIV, other STD/STI, or pregnancy prevention information relevant to LGBTQ+ youth (e.g., curricula or materials that use inclusive language or terminology).	R Square	.173
	B (SE)	.119 (.028)
	95% CI	.063–.174
	Sig.	*p* < .0001*
Practice 4: Encourage staff members to attend professional development on safe and supportive school environments for all students, regardless of sexual orientation, gender identity, or gender expression.	R Square	.378
	B (SE)	.135 (.019)
	95% CI	.098–.172
	Sig.	*p* < .0001*
Practice 5: Facilitate access to providers not on school property who have experience providing social and behavioral health services to LGBTQ+ youth.	R Square	.186
	B (SE)	.079 (.018)
	95% CI	.043–.114
	Sig.	*p* < .0001*
Practice 6: Facilitate access to providers not on school property who have experience providing sexual and reproductive health services to LGBTQ+ youth.	R Square	.165
	B (SE)	.082 (.020)
	95% CI	.043–.122
	Sig.	*p* < .0001*
All six evidence-informed practices	R Square	.364
	B (SE)	.090 (.013)
	95% CI	.064–.115
	Sig.	*p* < .0001*

* Significant at *p* < .0001.

Most schools made progress in implementing each EIP individually. The EIP with the lowest average implementation at the study's beginning was Practice 4: Encourage staff members to attend professional development on safe and supportive school environments for all students, regardless of sexual orientation, gender identity, or gender expression, with schools implementing on average 30% of practice elements. Practice 4 was also the EIP with the largest average change over time, with schools implementing nearly 90% of practice elements. The EIP with the highest average implementation at the study's beginning was Practice 1: Prohibit harassment and bullying based on a student's perceived or actual sexual orientation or gender expression (47%). Per Table 3, Practice 1 was also the EIP with the lowest average change, increasing to 82% by the end of the implementation period.

**Table 3:** Pre- and post-implementation adoption of evidence-informed practice elements in implementation condition schools.

Practice	Average Percent of Practice Elements in Place Pre-Implementation (Range)	Average Percent of Practice Elements in Place Post-Implementation (Range)	Average Change (Range)
Practice 1: Prohibit harassment and bullying based on a student's perceived or actual sexual orientation or gender expression.	46.8% (0%–90%)	82.1% (10%–100%)	35.3% (10%–70%)
Practice 2: Provide “safe spaces,” such as the school health office, counselor's office, designated classroom, or student organization, where LGBTQ+ youth can receive support from administrators, teachers, other school staff, or other students.	45.3% (13.3%–80%)	77.9% (26.7%–100%)	32.6% (−13.3%–73.3%)
Practice 3: Provide health education curriculum or supplemental materials that include HIV, other STD/STI, or pregnancy prevention information relevant to LGBTQ+ youth (e.g., curricula or materials that use inclusive language or terminology).	43.2% (0%–100%)	90.5% (0%–100%)	47.4% (−20%–100%)
Practice 4: Encourage staff members to attend professional development on safe and supportive school environments for all students, regardless of sexual orientation, gender identity, or gender expression.	30.5% (0%–80%)	89.5% (40%–100%)	58.9% (20%–100%)
Practice 5: Facilitate access to providers not on school property who have experience providing social and behavioral health services to LGBTQ+ youth.	45.1% (14.3%–100%)	78.2% (14.3%–100%)	38.3% (−14.3%–100%)
Practice 6: Facilitate access to providers not on school property who have experience providing sexual and reproductive health services to LGBTQ+ youth.	39.1% (0%–100%)	73.7% (14.3%–100%)	39.8% (0%–100%)
All six evidence-informed practices.	43.1% (10.2%–73.4%)	80.8% (28.6%–98%)	37.7% (8.2%–61.2%)

### Qualitative results: implementation of EIPs using the dynamic adaptation process

The quantitative analysis pointed to significant increases in EIP adoption compared to the baseline period; we qualitatively describe how the DAP facilitated EIP implementation from the perspectives of our participants and within the context of the EPIS framework below.

#### Exploration and preparation phases

Key activities during the early phases included convening and organizing IRTs; participating in the researcher-led multilevel assessment with feedback; initial training of IRTs; and coaching, ongoing assessment, and action planning.

**Convening and organizing of IRTs.** Each school was tasked with establishing an IRT of change champions—persons with influence in the school or knowledge and relationships necessary to accomplish action plan goals. Based on researchers' recommendations, school nurses were asked to lead IRTs comprising social workers, counselors, teachers, administrators, students, parents, and other school community members.

All participant types noted the value of diverse IRTs that enabled members to benefit from others' professional and personal expertise. One IRT member stated, “I appreciated being in this group because of the collaboration of all the professionals we all are.” A second explained, “Whenever we get together, everybody has good ideas about what they want to do…and…experiences that they've had or heard about.” This member, a school nurse, especially appreciated being privy to “the teacher perspective,” including “what they are seeing in the classroom” and “what's going on in conversations in their teacher meetings,” considering such information as crucial to action planning. Several IRT members also cited the value of having LGBTQ+ faculty and students on the teams, as they brought lived experience to the table when conducting assessments and designing implementation strategies.

Concerning IRT membership, participants widely observed that school health professionals, particularly nurses, had a platform to promote change regarding health education and service referrals. Counselors and social workers were also vital in adapting suicide prevention protocols to include LGBTQ+ students. Yet, due to resource scarcity, most schools had variable access to such professionals, with many splitting their time between multiple campuses and often stretched thin due to coverage gaps. In their stead, teachers and librarians assumed leadership roles in the IRTs, often taking responsibility for organizing GSAs and fashioning libraries into safe spaces for students to meet and access books and other materials on LGBTQ+ issues. One librarian described leveraging their professional role to support LGBTQ+ students: “I'm good at building relationships and I have the privilege to be the library media specialist because the libraries tend to pull in those students that maybe have been marginalized, so it's that safe space anyway.”

Teams with positive dynamics, i.e., productive collaboration with shared responsibility for action items, were especially effective. One IRT lead explained, “My gosh, they're [members are] so motivated. When we get together, it's like everybody's got great ideas, and [is] willing to help…. They're so self-motivated that if somebody's not there, [the work still happens].” IRTs that maintained member involvement met regularly and shared responsibility to continue progress in instances of turnover. In contrast, teams with a single person assuming most responsibility for EIP implementation retained fewer members while accomplishing less.

**Participating in the researcher-led multilevel assessments with feedback.** Once recruited into the study, school personnel took part in assessments on implementation readiness and climate, school-community collaborations, attitudes toward LGBTQ+ people, outer- and inner-context determinants, and perceived EIP implementation. The data were compiled into feedback reports disseminated to IRTs so they could address implementation facilitators and potential barriers when undertaking action planning. Common facilitators among schools included supportive administrators, strong LGBTQ+ champions, and community resources. Barriers included inadequate administrative support, knowledge and training deficits, and pragmatic concerns like time and staff availability.

The assessments also underscored outer-context issues that would influence how IRTs planned and interpreted the relative success of later efforts to implement EIPs. For instance, an IRT lead explained that although the school community was “open,” the broader community was less so, noting outspoken residents who were “closed-minded” and “prejudiced.” Another IRT lead described community members who antagonized school staff seeking to support LGBTQ+ students: “There were two teachers who received a note of Bible scriptures [that was] just very negative…. ‘Burn in hell’ kind of things.” A third IRT member mentioned a student dealing with an unsupportive family to illustrate the plight of some LGBTQ+ youth in the community:

The conversation among the teachers is that he is gay and struggling with it because of his home life. He comes from a ranching family, and ranchers are tough. He's the eldest boy…. Over the school year, he's had some disciplinary problems at school. I think it's all part of him trying to fit in…and…not knowing how to express himself.

The initial characterization of unsupportive communities and families and their adverse impacts on LGBTQ+ youth at home and in school persisted when the assessments were repeated in later years of RLAS. Feedback reports tapped such characterizations to highlight potential factors for IRTs to consider when prioritizing and planning for EIP implementation.

**Initial IRT training.** To support EIP implementation, the IRT members were asked to complete seven online webinars developed in collaboration with representatives from intermediary organizations in the CAP (Table 4). When transitions in IRT membership arose due to turnover, new members were invited to view the webinars to jumpstart their participation.

**Table 4:** Webinar topics and brief description of content.

Topic	Brief description of content
1. Dynamic Adaptation Process for Implementing Evidence-Based Strategies	Steps of this iterative implementation planning process, including member/partner engagement, initial assessment, action planning, implementation, and basic project management skills.
2. Leadership to Support Evidence-Based Strategies	Cultivation of transformational and implementation leadership skills and behaviors to encourage the use of new practices in school settings.
3. LGBTQ+ 101	Overview of sexual and gender minority people, including youth, and issues impacting their health.
4. Safe Zones Staff Training	Introduction to the purpose of the Safe Zones program and its main components; overview of resources to provide to LGBTQ+ youth in need; responsibilities, rights, and limitations of a Safe Zones volunteer; and launching a Safe Zones program in schools.
5. Transgender 101	Overview of definitions, concepts, and issues related to the health of transgender and gender-expansive people, including youth.
6. Bullying and Harassment Prevention	Overview of definitions and concepts of bullying, such as cyberbullying and harassment, connections between bullying, harassment, and bias, and intervention techniques for use in schools.
7. Suicide Prevention: Best Practices in School Settings	Facts and myths about suicide, warning signs, and intervention strategies for use in schools.

In the Preparation and Implementation Phases, researchers invited IRT leads to annual summer institutes to build capacity further through (1) fostering engagement and enhancing motivation among IRT leads, (2) reinforcing webinar content, and (3) modeling and practicing team-building activities IRT members could use to nurture relationships in larger school communities. Twenty-four IRT leads took part yearly. In evaluations and interviews, attendees described the institutes as venues for networking, exchanging ideas, sharing experiences, and learning from one another. Moreover, they fostered camaraderie among school professionals who felt isolated in their passion for supporting LGBTQ+ students due to the small or conservative nature of some schools. Several leads expressed gaining comfort in the knowledge that “I'm not alone in feeling alone” and being “encouraged about the community working on LGBTQ+ [issues].” In addition, the institutes showcased the expertise of local intermediary organizations specializing in adolescent health and LGBTQ+ issues, offering IRTs the chance to connect with their staff personally. One IRT lead emphasized the usefulness of the connections forged at the institutes, “I can call people and get them on the phone and say, ‘Hey! What do you think about this? Tell me about that?’ I called [an intermediary organization professional] and within 24 h I had an answer and a document I could email.”

**Coaching, ongoing assessment, and action planning.** Each IRT was assigned a coach with whom they met monthly. The coach's job was to support IRTs by offering guidance and facilitating access to resources, including consultation with intermediary organizations and training materials. Throughout the Preparation and Implementation phases, IRT members described forging trusting relationships through regular engagement with the coaches, viewing them as valued sources of information, encouragement, and empathy. To maintain such relationships, coaches had to balance motivating IRTs to increase activity levels to achieve goals and advising them to proceed at a slower pace to ensure goals were attainable.

As a first step, IRTs completed the structured collaborative assessment to measure EIP implementation progress and fidelity. Teams worked with coaches, reviewing data in the feedback reports produced from the original researcher-led assessments. When this assessment was first introduced, some administrators on the IRTs were reluctant to disclose that their schools were not already engaged in LGBTQ+ supportive activities or not doing so “perfectly,” as one principal admitted. Most administrators also presented with low awareness and knowledge of issues impacting LGBTQ+ student well-being, assuming that schools were doing their best to support LGBTQ+ youth. For example, many administrators disclosed that not knowing about instances of bullying meant they were likely not happening. Another principal explained, “We don't have a lot bullying or picking on or that kind of action that I'm aware of or have to address, so it leads me to believe that it's not just a huge issue here.”

Coaches, in turn, asked probing questions to encourage administrators' candor while striving to establish rapport with these individuals. Coaches and IRT members speculated that such administrators fretted about being “judged,” an idea repeatedly referenced in interviews. However, working with the coach on the structured collaborative assessment helped reduce such concerns while enhancing action plans. An IRT member discussed this process:

[The assessment] was very useful because there were questions we weren't aware of. When you talk about, “How’s our school?” “Are we very welcoming?” Right away, I would be like, “Absolutely! Yes!” She [the coach] provoked thought and gave other ideas. We're like, “Oh, we never thought of that. I guess we are lacking in that area.”

The assessment process was often perceived as “authoritative” by IRT members. It enhanced credibility when IRTs honed their goals and action plans for implementation. Coaches, by extension, were viewed as authoritative figures because of their fluency with the process and knowledge of EIPs. An IRT lead clarified, “That self-assessment document and having [our coach] guide us through [it] was amazing. He's an expert, and his guidance was incredible and helped us find out who we are and where we want to be.” Such perceptions of authority increased members' confidence when seeking buy-in from skittish or disengaged administrators.

The assessment process led IRTs to reflect on rolling out EIPs in their unique school contexts, encouraging a purposeful approach to planning. For example, IRT members had to contemplate if it was enough that their site's School-Based Health Center (SBHC) had a therapeutic support group for LGBTQ+ students after reviewing results. This prompted the IRT to consider the utility of also establishing a GSA to reach students who perhaps did not access SBHC services. These moments educated IRT members about the potential impact of changes at individual and organizational levels for action-planning purposes.

#### Implementation phase

Key IRT activities for this phase focused on organizing and engaging colleagues in professional development, adapting school contexts or EIPs to facilitate implementation, and monitoring and problem-solving implementation with coaching support.

**Prioritization of professional development to facilitate EIP implementation.** The DAP's flexibility allowed IRTs to approach implementation based on their local contexts, but patterns emerged across the sites. Training for school staff simultaneously reflected a key implementation strategy per the DAP and a focal EIP. Of all EIPs, professional development was a common early step. An IRT lead spoke of the need for greater education among school personnel: “The more knowledge we have, the more support I have, the more support I can give somebody else.” IRT members widely described training as foundational to implementing the other EIPs, creating buy-in while enabling more implementation champions to emerge.

The participation of school personnel in professional development reduced barriers, with IRT members and coaches asserting that training exposed staff to the realities of LGBTQ+ student struggles, which decreased complacency that could thwart EIP implementation. Offering all-staff professional development also provided opportunities to advertise the work of the IRTs, which attracted new members and circumvented the common pitfall of siloed implementation efforts. Moreover, training catalyzed new champions who assumed roles in crafting safe spaces by establishing GSAs and Safe Zone programs.

The IRT members suggested most colleagues were receptive to training. Still, they faced challenges organizing these events, including time constraints and resistance among a vocal minority of administrators and staff. As confirmed in coaching logs, IRTs commonly had difficulty reserving time on schoolwide or districtwide professional development calendars due to other state-mandated training or new training organized in response to crisis events (e.g., school shootings and the COVID-19 pandemic). Teacher and staff contracts also limited the professional development required for employment, meaning school personnel were not compensated for time spent attending “extra” training. The IRTs that engaged administrators well before the professional development calendar was set—often a full school year in advance—could usually secure a training spot upon the staff's return the week preceding fall instruction. This was a lesson the coaches sought to disseminate to all IRTs after the first training was organized in the early Implementation Phase. Administrative-level action also made it possible to designate time in staff schedules when school was in session. However, some staff refused to participate in training that they considered superfluous. In a few instances, complaints from unsupportive staff who did not represent most of their coworkers delayed or halted training. These complaints reportedly stemmed from anti-LGBTQ+ sentiment.

In addition to coaches, state and local intermediary organizations were key resources for implementation support, delivering in-person professional development for IRTs and school staff informed by the webinar series. The most popular trainings requested by schools were extended versions of LGBTQ+ 101, Transgender 101, and Safe Zones Staff Training. School staff responded favorably to collectively screening and discussing the award-winning documentary “I am Me,” which featured New Mexico youth sharing their experiences, including bullying, harassment, and discrimination in schools (67). An IRT lead described the importance of the screening, “[Our coach] came in and did the ‘I Am Me’ video, which I think is very important…. To me, that was one of the most powerful videos that I watched last year. I think that if we could share that with everybody, it would be a good starting point.” Suicide prevention training that included a focus on LGBTQ+ youth also increased staff comfort in engaging with students on the topic, offering practical tools for identifying and intervening appropriately with students in crisis, as well as shoring up school suicide prevention policies and practices.

Training was largely provided by LGBTQ+ people from intermediary organizations and, when possible, included school community representatives as presenters, strengthening relationships that allowed for continued expert consultation. Numerous IRT members across sites cited the invaluable assistance such organizations provided with training, as well as in establishing gender-inclusive restrooms on campus, developing and supporting gender support plans, and institutionalizing other inclusive policies and protections to create safe environments.

**Adaptation to facilitate EIP implementation.** The DAP supported IRTs in adapting school environments and practices to fit contexts and enable implementation. For instance, regarding LGBTQ+ inclusive materials in health education, some schools had informal or no health education, or relied on unqualified personnel (e.g., athletic coaches) and community organizations that encouraged abstinence to teach it. An IRT lead stated, “District policy is abstinence and things like that,” while a second observed, “We have pamphlets. We have posters. But there's no health curricula.” In such cases, schools altered their context by adopting curricula from other districts and establishing new health classes. However, such efforts were neither straightforward nor easy, leading some IRTs to de-prioritize changes for EIPs considered too ambitious to pursue. Speaking of health curricula adoption challenges, one IRT member shared: “Trying to address the sex ed curriculum, which is an absolute garbage fire on a good day…would require multiple teachers and probably the board and finding the resources…. [It would] also probably [involve] having unpleasant conversations with the teachers who teach it because of the awkwardness.” To impart ideas for overcoming such challenges, IRTs were provided training in implementing inclusive curricula through the summer institutes and consultation with CAP members with content-area expertise.

While in some instances it was necessary to change the school environments to make implementation possible, the EIPs were also the target of careful adaptation. The structured collaborative assessment delineated each EIP's core elements while enabling IRTs to determine their exact form. Adopting safe spaces, which involved organizing a social group where peers and adults could support LGBTQ+ youth, was a prime example of adapting practices. Some schools started groups openly labeled as GSAs. Anticipating community resistance, others changed the name to something less explicitly signaling LGBTQ+ identity. Elsewhere, libraries stayed open during lunch periods for informal gatherings, and SBHCs made confidential meeting spaces available for LGBTQ+ youth and their allies.

**Monitoring and problem solving.** Throughout the Implementation Phase, IRTs gathered local data and evaluated EIP implementation levels through the annually repeated structured collaborative assessments. Regular IRT meetings made it possible to document progress and determine barriers requiring attention in ongoing action planning, a core DAP mechanism.

When monitoring progress, several IRTs tapped student perspectives to enhance implementation by targeting issues relevant to LGBTQ+ students, rather than IRTs exclusively making decisions. For example, although the professional development EIP focused on school staff, some IRTs included youth collaborators in training presentations, which reportedly sharpened the content while making adults more receptive to participating. Students in one GSA shared strategies to engage their peers (e.g., organizing poster contests and panel discussions, changing backgrounds on library computers to feature suicide prevention hotlines, using social media) and helped revise a health education curriculum. Students elsewhere suggested visibility events for educating the school community, including a Day of (No) Silence. In this national student-led event, LGBTQ+ students and allies take a vow of silence to protest the effects of harassment and discrimination on sexual and gender minority people in schools (68). An IRT member described the event as particularly successful:

We had big banners and our activities director was really great because she had the banners put up in the central student area of the school. We tabled at lunch in that central area…. Then on the actual Day of Silence, they [the students] all had their little cards [to inform others that they were participating]. Then, after the end of the school day, we had a breaking the silence party, which a lot of people attended.

IRT members reported that students felt more empowered to get involved after recognizing their schools' efforts in undertaking LGBTQ+ specific interventions. Initially, school administrators and IRTs cited not knowing what LGBTQ+ students need as a barrier, as described earlier. However, when students felt safe enough to speak up, IRT members recognized them as key sources of insight into the on-the-ground realities LGBTQ+ youth face at school and in their communities. Indeed, IRT members, school administrators, and other staff disclosed during interviews that their unrealistic perceptions of how safe schools were for LGBTQ+ students were dispelled when given opportunities to hear from students about their lived experiences. For example, a student IRT member was able to speak directly and honestly to the principal, which another member described as a tense but crucial interaction:

The principal took it personally when [student name] explained that this school is not safe. [The student] was trying to talk and explain. The principal got defensive and talked over him…. That was a tense part. I don't think any of us was expecting that response. She caught herself. She knows that about herself, that she will get defensive…. That's not a bad thing about tension and that particular interaction. [The student] was able to continue telling her what he was trying to say. She calmed down enough to listen to him.

When asked about the experience, the student said, “Terrifying. Very. I really do think some of the things I said in the meeting opened a couple people's eyes.”

Much problem-solving centered on staff turnover. Turnover was common across all categories of school staff, reflecting a larger systemic issue in New Mexico. Turnover appeared least likely to impact the EIP concerning bullying and harassment policies, which, after adoption, remained in place. However, frequent turnover negatively influenced policy dissemination and enforcement. In one case, a study site almost completely replaced its faculty during the Implementation Phase. An IRT lead at that school recounted:

We had an election of new board members…. Those board members have made things unbearable for a lot of people…. We lost 50 staff…. Then, last week, we had basically the whole middle school eliminated, and eight teachers let go…. Then they said, this week, it's supposed to be the educational assistants [who] would be fired.

The school's board was disbanded shortly after this interview. This extreme example highlights how contexts could easily shift, requiring coaches to assess and troubleshoot challenges to IRT membership. However, as staff left schools, coaches often helped recruit IRT members and obtain support from new administrators. Coaches' knowledge of schools and relationships with staff made it possible to advise IRTs on strategies for sustaining their teams.

Implementation efforts requiring more than one school year to complete were the most vulnerable to failure due to staff turnover. IRTs dealt with this challenge by instantiating multiple leadership structures in their teams, typically identifying two leads rather than one, so teams would not be rudderless when one departed from the school. This “backup plan” allowed several IRTs to stay on track despite turnover in critical positions. Participants characterized this strategy as helpful in reducing stress and burnout among some IRT leads who worried about assuming the team's leadership responsibilities while juggling other job duties.

Many IRTs tried leveraging school and district leadership to troubleshoot challenges. Support (or lack thereof) from administrators at both levels greatly affected implementation progress, setting the tone for better or worse when introducing the EIPs. Implementation was aided when administrators like principals and assistant principals attended IRT meetings, vocally supported the EIPs among other staff, and took action to achieve implementation objectives. Such involvement enabled IRTs to achieve goals dependent on administration-level action, such as designating time in staff schedules for EIP-specific training (as described above). Supportive administrators at the school level were also proactive in addressing parental and community-based pushback. Additionally, administrative support in district offices boosted implementation efforts. In one school, for example, district administrators advocated for and passed a new anti-bullying policy protective of LGBTQ+ students and added it to student handbooks at the IRT's urging, despite the presence of community resistance. Elsewhere, IRT members parlayed respect from district administrators to gain support from principals of neighboring high schools for EIP implementation. These administrators then helped IRTs coordinate professional development at multiple schools and promoted district-wide LGBTQ+ cultural competency training for youth-serving behavioral health and medical providers.

Nevertheless, advancing EIPs requiring district leadership involvement (e.g., enabling access to LGBTQ+ inclusive sexual and reproductive healthcare services in underserved settings) and when practices implicated the surrounding community (e.g., effectuating provider linkages) sometimes met with challenges. Some IRT members responded by working around seemingly unchangeable policies governing schools when district leadership was unwilling to implement formal protocols or foster connections to other resources to facilitate the specific support students needed. In one example, an IRT lead and nurse at a school without an SBHC described “walking a line” by informally directing students without district approval to state-operated healthcare clinics, called “Public Health,” to obtain care for sexual and reproductive health concerns. The lead stated, “Public Health is one of my major resources for students…. Now, for LGBT, it's STIs and pregnancy. They can go without the parent knowing and be seen.”

Regardless of how involved district and school administrators were with the EIPs, a key lesson learned by IRTs was to keep them abreast of their efforts or risk reprimand and other consequences. One IRT learned this lesson after opting to “fly under the radar” and “ask forgiveness [rather] than permission” for pursuing items on its action plan. Controversy arose when the IRT invited local health educators to share a curriculum inclusive of LGBTQ+ people in health classes without the principal's consent. Reportedly feeling disrespected by decisions made by the IRT without his knowledge and anti-LGBTQ+ bias in the school, the principal disinvited the health educators, barred the curriculum, and stalled the IRT's progress.

#### Sustainment phase

The DAP's emphasis in the Sustainment Phase was on keeping EIPs in place. However, the March 2020 public health response to the COVID-19 pandemic in New Mexico coincided with the third year of the Implementation Phase. It emerged as a dominant force throughout the Sustainment Phase until schools transitioned from virtual to in-person learning almost one year later. During this period, school personnel, including IRT members, struggled to perform their regular work, let alone maintain progress in implementing the EIPs. Despite challenges, most IRTs provided support through creative means, spurred on by growing fears about student mental health, isolation, dangers facing LGBTQ+ youth (e.g., violent homes, identity concealment, maladaptive coping), and loss of family and community members. An IRT member shared, “Our students just suddenly got cut off, and they're out there floating in space without any of us.… It seems like a bad time to turn our backs on all the kids.” Their adaptations to the pandemic as a major outer-context determinant included hosting GSAs online with varying success; navigating communication challenges by leveraging electronic connectivity (e.g., emails, instant messages); and using CAP guidance to vet and integrate enhanced resource lists with information on LGBTQ+ affirming health providers, mental health hotlines, and other resources into school websites and learning platforms. Other adaptations included adding LGBTQ+ affirming symbols to email signatures and video backgrounds and holding virtual “drop-in” hours. The relationships forged between IRTs and intermediary organizations during the Implementation Phase were pivotal to maintaining progress. The IRTs could rely on these ties in the Sustainment Phase without needing a coach.

### Joint display of quantitative and qualitative findings

Table 5 focuses on the intersection of quantitative and qualitative data related to EIP adoption outcomes, underscoring the DAP's importance in leveraging facilitators and addressing implementation barriers, including through problem-solving and adaptation.

**Table 5:** Joint display of quantitative and example qualitative findings.

Quantitative Findings	Example Qualitative Findings^a^
**Practice 1:** Prohibit harassment and bullying based on a student's perceived or actual sexual orientation or gender expression	
• Significant increase in adoption of policies and practices that prohibit bullying based on a student's perceived or actual sexual orientation or gender expression.	• Bullying and harassment policies were often controlled at the district level and outside of the direct sphere of IRT influence. • Assessments revealed that having such policies did not mean they were enforced or that school personnel, including administrators, recognized that bullying and harassment of LGBTQ+ students occurred on campus. • IRTs that progressed in adopting and raising awareness of such policies made it a point to engage district leadership and dispel beliefs that on-campus bullying and harassment were not problems, including among school administrators. • Newly passed state legislation in 2020 required schools to implement LGBTQ+ inclusive bullying policies, an outer-context development with potential to reinforce implementation efforts in the inner context. • As part of problem-solving with IRTs, coaches and the CAP produced guidance documents to aid schools in responding to the policy change.
**Practice 2:** Provide “safe spaces,” such as the school health office, counselor's office, designated classroom, or student organization, where LGBTQ+ youth can receive support from administrators, teachers, other school staff, or other students	
• Significant increase in adopting “safe spaces.”	• Most IRTs prioritized starting new or strengthening existing programs, i.e., Genders and Sexualities Alliances and Safe Zones, as high-impact steps to kick-start culture and climate change in schools. • Professional development for school personnel was useful in attracting adult participation in starting or strengthening existing programs. • IRTs supported students in starting Genders and Sexualities Alliances, ranging from small lunch gatherings to robust and well-publicized clubs. • IRTs formed Safe Zone teams to foster supportive environments. Safe Zone volunteers received training and displayed symbols on doors and walls (and later online) to signify their availability for support. • Students supported IRT efforts, including sponsoring school-wide art competitions with LGBTQ+ themes to signify inclusive environments. • Creating informal safe spaces represented an important adaptation pursued by the IRTs, with librarians procuring and highlighting LGBTQ+ affirming books and materials. • IRTs leveraged connections forged through the summer institutes and other training, partnering with intermediary organizations to cultivate safe spaces for gender-diverse students, including by advocating for and adopting the use of gender support plans and gender inclusive restrooms.
**Practice 3:** Provide health education curriculum or supplemental materials that include HIV, other STD/STI, or pregnancy prevention information relevant to LGBTQ+ youth (e.g., curricula or materials that use inclusive language or terminology)	
• Significant increase in the adoption of inclusive health education curricula and materials.	• Schools and districts lacked guidance and resources to abide by state government-issued content standards and varied widely in curricula. • Assessments revealed that school personnel without appropriate training often taught health education (e.g., athletic coaches), and school administrators were often unaware of the material covered in health-related classes. Staff turnover also led to a lack of content uniformity. • Fearing community pushback, individual educators and administrators were commonly reluctant to make curricula or other educational materials inclusive. • As part of problem-solving with IRTs, coaches and the CAP produced health education guidance documents and other school professionals to assess curricula, develop skills, and access resources and training. • The pervasive lack of established curricula for health education in most schools, combined with staff discomfort and insufficient skillsets concerning the subject matter, led IRTs to adapt both contexts and practice to accommodate implementation.
**Practice 4:** Encourage staff members to attend professional development on safe and supportive school environments for all students, regardless of sexual orientation, gender identity, or gender expression	
• Significant increase in the adoption of professional development for staff.	• IRT efforts to organize and deliver professional development for staff, enhanced buy-in and support for implementing other practices centered on creating a safe and supportive environment for LGBTQ+ students. • IRTs engaged in substantial problem-solving with their coach to make professional development available, due to time constraints, competing demands, and resistance from colleagues and communities. • IRTs overcame challenges to professional development through engagement with school and district leadership, as well as intermediary organizations that offered training on practice-related topics. • Building relationships with intermediary organizations made the sustainment of this practice possible since ongoing training was not reliant on either coach or CAP involvement.
**Practices 5 and 6:** Facilitate access to providers not on school property who have experience providing (a) social and behavioral health services and (b) sexual and reproductive health services to LGBTQ+ youth	
• Significant increase in the adoption of practices for facilitating access to (a) social and behavioral health and (b) sexual and reproductive health services.	• The lack of direct service providers in underserved communities undermined IRTs' ability to facilitate student access to services. • SBHCs on some school campuses provided services related to both practices but struggled with restricted operating hours and staff turnover. • School personnel would make informal referrals to state-operated health clinics for confidential sexual and reproductive health services. • As an adaptation within underserved communities, IRTs organized LGBTQ+ specific professional development for local providers. • As part of problem-solving with IRTs, coaches and the CAP developed a guide to help school staff vet local providers, boosting confidence in directing LGBTQ+ students to qualified care.
**All 6 Practices**	
• Significant increase in the adoption of all six evidence-informed practices.	• Professional development fulfilled Practice 4 and built buy-in for other practices, bolstering Practice 2 through training on safe spaces and Practices 1, 3, 5, and 6 via education on health disparities and specialized support needs. • Practices 3, 5, and 6 often had corresponding action steps ensuring that resource lists and educational materials on health were inclusive of LGBTQ+ youth. • IRTs monitored the impact of practice implementation to adjust action plans and improve practice uptake and effectiveness.

^a^ Abbreviations in alphabetical order: CAP, community-academic partnership; DAP, dynamic adaptation process; IRT, implementation resource team; SBHC, school-based health center.

## Discussion

The DAP is a participatory implementation science model that can address health equity for marginalized populations by enabling the uptake of practices to advance well-being. When paired with the EPIS framework and bolstered by a CAP, it encourages consideration of outer- and inner-context factors and innovation characteristics likely to influence implementation and a staged approach for assessment, convening IRTs to manage EIP implementation, preparing school personnel to support EIPs, and using feedback data to guide appropriate adaptation and monitor progress (27, 69). Researchers have called for a deeper understanding of how the DAP is operationalized through engagement with diverse partners across the implementation process (41). Our mixed-methods study of processes and outcomes produced through the DAP answers this call by offering a novel example for applying this multifaceted implementation strategy to tackle LGBTQ+ health disparities in schools. To our knowledge, RLAS is the first statewide study to use the DAP to (1) implement a suite of EIPs vs. a single practice, (2) support the adoption of new practices in an education system rather than a healthcare or human service system, and (3) facilitate non-clinical interventions to reduce stigma and discrimination for a health equity population at a large scale. Whereas other studies highlight only a selection of DAP's key components or provide little insight into the model's operationalization (28, 29, 70), our analysis illuminates the utilization of all components through the four EPIS phases, assesses their contributions to EIP adoption, and elucidates how implementers in real-world settings adapt contexts and practices to ensure success when issues of innovation fit paramount are owing to societal discrimination and stigma (42). Rather than assume a simple or linear path to positive adoption outcomes, we sought to unpack the black box of applying the DAP under sometimes trying real-world circumstances that can curtail implementation (66), thus making a valuable contribution to implementation science examinations of adaptation and scale out of practices for health equity goals in complex sociocultural systems like schools (69, 71).

Per the DAP, schools formed IRTs that gathered and reviewed local data regarding their status vis-à-vis the EIPs to inform action planning and adaptation. The initial and ongoing assessments of DAP helped IRT members appreciate the importance of context when developing, monitoring, and adjusting plans. The ongoing assessments and action planning were useful for (1) anchoring teams to stay focused on remaining work and (2) socializing new IRT members by providing a common understanding of the EIPs and barriers requiring troubleshooting. In most other clinically-based studies of the DAP, the formation of IRTs is not explicitly discussed (31, 43). Minimal details on how they were deployed are provided (29). Healthcare providers have also individually received feedback on implementation and adaptation from intervention experts, with adaptations identified before implementation and then retrospectively analyzed afterward (34). For RLAS, having IRTs made it possible to ensure local context drove implementation and adaptation. The teamwork required within the DAP also nurtured a cadre of champions who could support one another during the Implementation Phase.

Given the time it takes to make changes in schools, a notable feature of the DAP is the ongoing problem-solving of issues occurring in inner and outer contexts, owing to the iterative nature of implementation, feedback, and adaptation alongside the cultivation of champions. We found the DAP promoted implementation leadership that enabled problem-solving in multilevel school contexts, including in conservative social settings where school professionals were sometimes reticent to introduce practices to improve LGBTQ+ student well-being (9, 72). The EPIS framework also identifies support from leaders at all levels as critical to implementation success (10, 15). Our qualitative investigation revealed that district-level leadership support at the outer context empowered IRTs to implement new EIPs, as in the example of the school where district administrators advocated for and passed a new anti-bullying policy explicitly protective of LGBTQ+ students, integrating it into student handbooks despite local resistance. School administrators influenced the terms of affirming LGBTQ+ students and enabling the introduction of new practices within the inner context. Per the DAP, the IRTs benefited most when administrators actively supported their efforts by doing basic things like regularly making time for IRT meetings and activities, vocalizing their support for the EIPs, and taking action to achieve implementation objectives.

Coaching, another key DAP component, was vital to implementing the EIPs with fidelity in under-resourced school environments rife with competing demands. Our use of the DAP included more intensive coaching and technical assistance than in prior published work, such as the first DAP study on implementing a home visitation program (27). Here, the intervention purveyor coached implementers to support fidelity to this evidence-based intervention. In contrast, RLAS coaches nurtured capacity building to support EIP adoption and encouraged variability in EIP implementation when fidelity to core functions was maintained. With CAP support and capitalizing on their imputed authority, coaches helped IRTs develop collaborative ties with local experts and organizations, making training and consultation readily available. In addition, the condition of the statewide implementation context—one marked by universally high turnover (47)—also prompted the coaches to assume valuable but unanticipated roles in recruiting for the IRTs and advocating before new school leadership. The research team did not originally envision these roles for coaches, yet coaches' regular engagement and relationships with the IRTs positioned them to respond to such challenges.

Professional development through webinars, summer institutes, and in-person training propelled DAP functioning. It contributed to EIP adoption by increasing IRT member knowledge and skills, building buy-in among the wider school staff, and creating favorable conditions for implementation. Our prior work clarifies that impactful professional development should empower all staff to increase awareness of LGBTQ+ terminology, comfort in using neutral and gender-inclusive language, and knowledge of practical ways schools can address poor health outcomes for LGBTQ+ students (72–74). Effective professional development will likely invest staff in making changes, ranging from personal language use to school policies. One-time, stand-alone training will not result in lasting change within schools (75). Onboarding processes for new staff and recurring (e.g., yearly) training for all school personnel, including for support staff (e.g., custodians, bus drivers, cafeteria workers), may be instrumental in creating a safe and supportive school culture for LGBTQ+ students, especially when training is dynamic (76, 77). Widespread professional development among staff also increases the likelihood that students will have support and safety wherever they are or with whomever they interact (77).

Given the importance of coaching and leadership in supporting implementation and sustainment in contexts of high turnover, low resources, and resistance to change, future work on LGBTQ+ supportive EIPs could aim to build such capacity at district and state levels to buffer against inevitable challenges hampering such initiatives. For example, the Interagency Collaborative Team strategy based on the DAP featured a “seed team” spanning multiple agencies that built local coaching capacity to ensure continued program implementation and sustainment without ongoing reliance on the intervention purveyor (27, 78, 79). Using a seed team model to embed coaching capacity and set expectations for EIP implementation for school administrators and other personnel in the inner context can provide the footing to sustain the implementation progress made by IRTs and create new IRTs in schools lacking them. The seed team can also help with readiness assessments, enable access to training and resources, connect school personnel to local intermediary organizations, and assist with monitoring implementation.

As champions of EIP implementation traversing outer and inner contexts, a seed team at state and district levels could cultivate implementation leadership among their colleagues while promoting leadership alignment across the state, districts, and schools to overcome multilevel barriers to the uptake and sustainment of innovations. By encouraging such alignment, the IRTs would be better able to address aspects of EIP implementation efforts falling outside the domain of individual school-level control, including district policies or the content and quality of health education curricula, or that require higher-order intervention, such as addressing the absence of medical and behavioral health providers in underserved states like New Mexico. They would also be well-positioned to influence or stay abreast of state policies likely to shape EIP implementation and convey this information in an actionable form to school-based IRTs.

States and districts are responsible for addressing school staff turnover (47, 80), a problem that can hamper the successful implementation of most initiatives (13). In our study, the DAP encouraged recruiting new IRT members as needed, accommodating turnover to some extent. In understaffed schools, however, turnover means school personnel must assume greater responsibilities to maintain school operations, possibly making some staff reluctant to join an IRT, diverting current members' attention from DAP activities, and sapping momentum for implementation. Tackling turnover requires a concerted policy response by districts and states, including creative workforce development and retention initiatives beyond raising salaries (80).

Finally, the DAP may be well-suited to implement the types of EIPs supported by RLAS because of its central focus on adaptation-preserving function while allowing flexibility in form. Recent implementation studies clarify the need to add to or shift our referent for the fidelity of interventions toward considering their core functions or the purposes interventions serve, rather than only the form or packaging of those functions (40, 81–83). This shift frees interventionists and implementation scientists to adjust forms to be more acceptable, appropriate, and feasible for communities. RLAS demonstrates that for interventions addressing health disparities rooted in stigma, adapting to fit contexts while making potentially unwelcome changes more acceptable, appropriate, and feasible improves chances for interventions to fulfill their functions.

Our study has limitations. It describes the use of the DAP process to implement EIPs for enhancing LGBTQ+ health equity in one state in the Southwestern United States, so our findings may not be generalizable to sites elsewhere. Overall, participants in the study were interested and committed to implementing LGBTQ+ supportive EIPs. Schools with uninvested administrators and personnel were less likely to participate in these change efforts or data collection. Quantitative analyses of EIP adoption were limited to data drawn only from implementation schools, so we cannot compare conditions over time.

Despite its limitations, RLAS illustrates the DAP's effectiveness as a multifaceted implementation strategy for adapting and adopting a suite of EIPs to promote the health of a stigmatized population. For the DAP to succeed, it may be essential to create a support infrastructure, including leveraging CAPs and embedding coaching and consultation capacity in state health and education departments or districts. Professional development can scaffold the DAP. This infrastructure should facilitate initial and ongoing staff training and link schools to training opportunities and LGBTQ+ intermediary organizations. States and districts must make concerted efforts to reduce school turnover (80), which can compromise processes like the DAP and the implementation and scale-out of interventions (69) for supporting safety and wellness in school communities, especially for students affected by health disparities (9).

## Data Availability

The de-identified data supporting the conclusions of this article will be made available by the authors upon reasonable request.
