# Supplementary material for: Leveraging the dynamic adaptation process to address LGBTQ+ health equity in New Mexico high schools
Source: Front Health Serv. 2025 Jun 3;5:1499508. doi: 10.3389/frhs.2025.1499508 (PMC12170598; doi:10.3389/frhs.2025.1499508)
Supplement: Supplementary file 1 [file Datasheet1.pdf]

RLAS
Reducing LGBTQ Adolescent Suicide

ASSESSMENT OF SCHOOL PRACTICES TO REDUCE LGBTQ+ ADOLESCENT SUICIDE

INTRODUCTION

The Centers for Disease Control and Prevention (CDC) identifies six evidence-informed practices that are likely to benefit the health and well-being of high school students, particularly those who might identify as lesbian, gay, bisexual, transgender, and questioning or queer (LGBTQ+).

Practice 1:	Prohibit harassment and bullying based on a student's perceived or actual sexual orientation or gender expression.
Practice 2:	Provide "safe spaces," such as the school health office, counselor's office, designated classroom, or student organization where LGBTQ+ youth can receive support from administrators, teachers, other school staff, or other students.
Practice 3:	Provide health education curriculum or supplemental materials that include HIV, other STD/STI, or pregnancy prevention information that are relevant to LGBTQ+ youth (e.g., curricula or materials that use inclusive language or terminology).
Practice 4:	Encourage staff members to attend professional development on safe and supportive school environments for all students, regardless of sexual orientation, gender identity, or gender expression.
Practice 5:	Facilitate access to providers not on school property who have experience providing social and behavioral health services to LGBTQ+ youth.
Practice 6:	Facilitate access to providers not on school property who have experience providing sexual and reproductive health services (SRHS) to LGBTQ+ youth.

This assessment tool is designed to help your Implementation Resource Team (IRT) determine the degree to which your school may be implementing each of the six practices. We encourage the full IRT to participate in completing this assessment tool. Your IRT will be able to use the information provided through this tool to determine a minimum of two evidence-informed practices to focus on for this coming school year.

An RLAS Coach is available to assist the team in using the tool and answer any questions regarding the content of the assessment. Below is a list of abbreviations and a glossary of terms included in the tool. You may not know all the answers, but do your best. If something is not applicable, you can leave it blank and/or leave notes in the comments.

Please note that information from this assessment will be treated as confidential information and used primarily for planning and evaluation purposes. The individual results of this assessment will not be shared with persons outside of the RLAS Research Group.

LIST OF ABBREVIATIONS

CDC – Centers for Disease Control and Prevention
GSA – Gay Straight Alliance or Genders and Sexualities Alliance
HIV – Human Immunodeficiency Virus
IRT – Implementation Research Team
LGBTQ+ – Lesbian, Gay, Bisexual, Transgender, and Questioning/Queer
RLAS – "Reducing LGBTQ+ Adolescent Suicide"
SRHS – Sexual and Reproductive Health Services
STD/STI – Sexually Transmitted Disease/Sexually Transmitted Infection

GLOSSARY OF KEY TERMS USED IN THIS DOCUMENT

Gender Expression: Gender expression refers to all of the external characteristics and behaviors that are defined in society as either masculine or feminine, such as dress, grooming, mannerisms, speech patterns, and social interactions. This is what an individual chooses to show the world.

Gender Identity: Gender identity refers to a person's deeply felt identification as a man, woman, or other gender. This may or may not correspond to the sex assigned to them at birth.

Gender Inclusive and/or Gender Neutral Language: Inclusive and neutral language does not make assumptions about an individual's gender identity, gender expression, or sexual orientation. Examples can include asking about someone's "significant other" or "person you're dating" instead of saying "boyfriend" or "girlfriend," saying "students" instead of "boys and girls," or simply using individuals' names.

Gender Pronouns: Gender pronouns are words that refer to people without using their names. Common gender pronouns include she/her/hers, he/him/his, and they/them/theirs. (Note that using they/them/theirs as a singular pronoun is acceptable.)

Safe Space: In reference to Practice 2 of the CDC's six evidence-informed practices, "safe spaces" refer to intentional efforts to make students' spaces welcoming and inclusive to LGBTQ+ youth, such as the school health office, counselor's office, designated classroom, or student organization where LGBTQ+ youth can receive support from administrators, teachers, other school staff, or other students. This can often include restrooms and policies around gender that make the school environment accessible to LGBTQ+ youth.

Safe Zones: Safe Zones is an intentional program that creates a safer, more welcoming, and inclusive environment for all students, but especially marginalized students such as LGBTQ+ students, students of color, students with disabilities, and undocumented students. In an effective Safe Zone Program, school staff receive training about the issues of marginalized populations and referral resources. Trained school staff display signs in their office or classroom, declaring the space a "safe zone."

Sexual Orientation: Sexual orientation refers to a person's physical or emotional attraction to someone. This can be toward the same and/or different gender.

School-Based Health Center (SBHC): An SBHC is a health clinic located on or near school property that functions separately from and in cooperation with the school nurse's office.

Assessment of School Practices to Reduce LGBTQ+ Adolescent Suicide

SITE INFORMATION	
**School:**	**Date(s) of completion:**
**Name(s)/Position(s) of Implementation Resource Team members completing assessment:**	
**Name of Coach(es) helping to complete this assessment:**	

Please indicate the most appropriate answer. Feel free to clarify any answer in the "other comments" box.

__Y__ – (Yes: this item is currently in place)
__N__ – (No: this item is not in place)
__U__ – (Undetermined: unable determine if the item is currently in place or is not.)

1. <u>PRACTICE 1:</u> Prohibit harassment and bullying based on a student's perceived or actual sexual orientation or gender expression.			
**SCHOOL DISTRICT**			
a. District maintains a <u>non-discrimination policy</u> that is explicitly inclusive of students of diverse sexual orientation and/or gender identity or gender expression.	Y	N	U
b. District maintains a <u>policy against bullying and harassment</u> that is explicitly inclusive of students of diverse sexual orientation and/or gender identity or gender expression.	Y	N	U
c. District has a systematic process for annually disseminating the policy described above to all <u>teaching and administration</u> staff.	Y	N	U
d. District has a systematic process for annually disseminating the policy described above to <u>support staff</u> with direct contact with students.	Y	N	U
e. District maintains a statement of student and staff rights and responsibilities promoting behaviors consistent with values of diversity, inclusion, and respect for all persons.	Y	N	U
f. District maintains an advisory group that monitors progress towards safe and supportive environment for LGBTQ+ students.	Y	N	U
g. District has an established mechanism that is implemented in all schools for collecting feedback about bullying and harassment incidents.	Y	N	U

SCHOOL SITE			
h. School administration conducts annual activities to disseminate district-level policies that support a safe and supportive environment for LGBTQ+ students.	Y	N	U
i. School administration elicits feedback from students or school staff regarding the implementation and effectiveness of policies intended to maintain a safe and supportive environment for LGBTQ+ students.	Y	N	U
j. School administration consistently follows up regarding action taken with parties involved in violations of harassment and bullying policy.	Y	N	U

PRACTICE 1 SUMMARY
**On a scale of 1 to 5, how would this IRT rate the school's CURRENT level of implementation of Practice 1?**<br>① *no implementation*<br>② *a little implementation*<br>③ *some implementation*<br>④ *nearly full implementation*<br>⑤ *full implementation*
**What is working out best to support the implementation of Practice 1?**
**What are some opportunities to better support the implementation of Practice 1?**
**Other comments:**

2. PRACTICE 2: Provide "safe spaces" such as the school health office, counselor's office, designated classroom, or student organization where LGBTQ+ youth can receive support from administrators, teachers, other school staff, or other students.			
**SAFE SPACES**			
a. At least one club or group tailored to LGBTQ+ students is present in the school, e.g., Gay Straight Alliance/Genders and Sexualities Alliance (GSA) or other groups/clubs.	Y	N	U
b. The school participates in a Safe Zone Program, or similar program, in which staff display posters, stickers, etc., indicating that they are a trained resource person and/or that their office or classroom is a safe and welcoming space for LGBTQ+ students.	Y	N	U
c. School administration ensures that all school staff are aware of the school's participation in the Safe Zone Program or similar program.	Y	N	U
d. Training for the Safe Zone Program, or a similar program, is available for staff interested in volunteering.	Y	N	U
**OUTREACH AND COMMUNICATIONS**			
e. Notices about LGBTQ+-focused school activities (GSA meetings, etc.) are posted in multiple locations throughout the school.	Y	N	U
f. Notices about LGBTQ+-focused resources (school-based or community) are posted in multiple locations throughout the school.	Y	N	U
g. Materials are publicly displayed that offer representations of LGBTQ+ people and/or include language about diversity and inclusion of all students and staff, regardless of actual or perceived sexual orientation or gender.	Y	N	U
**POLICIES AND PRACTICES ON GENDER**			
h. Single-unit bathrooms are available for any student or staff who expresses a concern about safety or privacy without excluding persons with concerns related to gender identity, gender expression, or sexual orientation.	Y	N	U
i. School staff use gender-inclusive and gender-neutral language as a standard practice during classroom instruction and when interacting with students and other school staff.	Y	N	U
j. Both opposite-gender and same-gender couples are welcome at school dances and events.	Y	N	U
k. Dress codes and uniform requirements (including those for physical education) permit students to wear clothes that align with their gender expression.	Y	N	U
l. Physical education and sports programs include co-ed teams to maximize opportunities for LGBTQ+ students to participate. Examples include co-ed dance groups or co-ed non-contact sports teams, such as volleyball or golf.	Y	N	U
m. School administration has a process in place to support student requests for preferred gender pronouns and chosen names, including updating student information systems.	Y	N	U
n. School staff consistently refer to students by their preferred gender pronouns and chosen names.	Y	N	U

PRACTICE 2 SUMMARY
**On a scale of 1 to 5, how would this IRT rate the school's CURRENT level of implementation of Practice 2?**<br>① *no implementation*<br>② *a little implementation*<br>③ *some implementation*<br>④ *nearly full implementation*<br>⑤ *full implementation*
**What is working out best to support the implementation of Practice 2?**
**What are some opportunities to better support the implementation of Practice 2?**
**Other comments:**

3. PRACTICE 3: Provide health education curriculum or supplemental materials that include HIV, other STD/STI, or pregnancy prevention information that are relevant to LGBTQ+ youth (e.g., curricula or materials that use inclusive language or terminology).

SCHOOL			
a. The health education curriculum materials go beyond "abstinence-only" to include comprehensive sex education.	Y	N	U
b. The health education curriculum or supplemental materials include HIV information with language that is inclusive of LGBTQ+ students.	Y	N	U
c. The health education curriculum or supplemental materials include other STD/STI information with language that is inclusive of LGBTQ+ students.	Y	N	U
d. The health education curriculum or supplemental materials include pregnancy prevention information with language that is inclusive of LGBTQ+ students.	Y	N	U
e. The health promotion materials available at the school health office include health information inclusive of LGBTQ+ students.	Y	N	U

PRACTICE 3 SUMMARY

On a scale of 1 to 5, how would this IRT rate the school's CURRENT level of implementation of Practice 3?

① *no implementation*

② *a little implementation*

③ *some implementation*

④ *nearly full implementation*

⑤ *full implementation*

What is working out best to support the implementation of Practice 3?

What are some opportunities to better support the implementation of Practice 3?

Other comments:

4. PRACTICE 4: Encourage staff members to attend professional development on safe and supportive school environments for all students, regardless of sexual orientation, gender identity, or gender expression.			
a. The school or district offers at least one annual training that addresses how school staff can contribute to fostering a safe and supportive environment for LGBTQ+ students.	Y	N	U
b. The school or district offers additional optional training (in-person or online) addressing how school staff can contribute to fostering a safe and supportive environment for LGBTQ+ students; such training is promoted to school staff annually.	Y	N	U
c. Feedback mechanisms are in place for the school administration to collect ideas from school staff for training topics and content areas specific to LGBTQ+ students, their needs, and their safety.	Y	N	U
d. Resources are disseminated to all school staff to assist them in responding to the needs of LGBTQ+ students (e.g., LGBTQ+ specific and/or inclusive helplines, online resources, community groups, etc.).	Y	N	U
e. School administration communicates to all school staff the expectations for a minimum demonstrated proficiency in responding to bullying and harassment of all students, including LGBTQ+ students, and participation in the creation of an inclusive environment.	Y	N	U

PRACTICE 4 SUMMARY

On a scale of 1 to 5, how would this IRT rate the school's CURRENT level of implementation of Practice 4?

① *no implementation*

② *a little implementation*

③ *some implementation*

④ *nearly full implementation*

⑤ *full implementation*

What is working out best to support the implementation of Practice 4?

What are some opportunities to better support the implementation of Practice 4?

Other comments:

**5. PRACTICE 5: Facilitate access to providers not on school property who have experience providing social and behavioral health services to LGBTQ+ youth.**			
a. The school has an SBHC prepared to provide affirmative and inclusive behavioral health services to LGBTQ+ youth.	Y	N	U
b. The school has a referral guide (paper-based [posters, palm cards, tear-off sheets] or electronic [database, website, mobile app]) that lists social service and behavioral health providers in the community, including those experienced in working with LGBTQ+ youth.	Y	N	U
c. The school has staff that can facilitate access to community-based social service and behavioral health providers.	Y	N	U
d. The school has marketing materials (e.g., posters) for community-based behavioral health services (not SBHCs) that are welcoming and inclusive of LGBTQ+ students.	Y	N	U
e. The school has policies and/or procedures in place to facilitate referrals for community-based behavioral health services.	Y	N	U
f. The school has a mechanism to follow up on referrals for behavioral health services	Y	N	U
g. There is management and oversight of the referral system for facilitating access to community-based behavioral health services.	Y	N	U

**PRACTICE 5 SUMMARY**
**On a scale of 1 to 5, how would this IRT rate the school's CURRENT level of implementation of Practice 5?**<br>① *no implementation*<br>② *a little implementation*<br>③ *some implementation*<br>④ *nearly full implementation*<br>⑤ *full implementation*
**What is working out best to support the implementation of Practice 5?**
**What are some opportunities to better support the implementation of Practice 5?**
**Other comments:**

**6. PRACTICE 6: Facilitate access to providers not on school property who have experience providing sexual and reproductive health services (SRHS) to LGBTQ+ youth.**			
a. School staff can refer LGBTQ+ youth to an SBHC for sexual and reproductive health services (SRHS).	Y	N	U
b. The school has a referral guide (paper-based [posters, palm cards, tear-off sheets]) or electronic [database, website, mobile app]) resource that lists sexual and reproductive health providers, including those experienced in working with LGBTQ+ youth.	Y	N	U
c. The school has staff who can facilitate access to community-based SRHS.	Y	N	U
d. The school has marketing materials (e.g., posters) for school and community-based SRHS that are welcoming and inclusive of LGBTQ+ students.	Y	N	U
e. The school has policies and/or procedures in place to facilitate referrals for community-based SRHS.	Y	N	U
f. The school has a mechanism to follow up on referrals for SRHS referral system.	Y	N	U
g. There is management and oversight of the referral system to facilitate access to SRHS in the community.	Y	N	U

PRACTICE 6 SUMMARY

On a scale of 1 to 5, how would this IRT rate the school's CURRENT level of implementation of Practice 6?

① *no implementation*

② *a little implementation*

③ *some implementation*

④ *nearly full implementation*

⑤ *full implementation*

What is working out best to support the implementation of Practice 6?

What are some opportunities to better support the implementation of Practice 5?

Other comments:

PLANNING

1. Consider the following evaluation criteria for each of the six practices:

- ***Importance:*** *How important is this action to my school?*
- ***Cost:*** *What are some of the costs of planning and implementing this action? How expensive would it be?*
- ***Time:*** *How much time and effort would it take to implement this action at my school?*
- ***Enthusiasm:*** *How enthusiastic would the school community be about implementing this action?*
- ***Feasibility:*** *How difficult would it be to implement this action in my school?*

For each Practice, please rank each criteria item on a scale of 1-5. (1=low, 5=high).	*Importance*	*Cost*	*Time*	*Enthusiasm*	*Feasibility*
**Practice 1:** Prohibit harassment and bullying based on a student's perceived or actual sexual orientation or gender expression.	① ② ③ ④ ⑤	① ② ③ ④ ⑤	① ② ③ ④ ⑤	① ② ③ ④ ⑤	① ② ③ ④ ⑤
**Practice 2:** Provide "safe spaces" such as the school health office, counselor's office, designated classroom, or student organization where LGBTQ+ youth can receive support from administrators, teachers, other school staff, or other students.	① ② ③ ④ ⑤	① ② ③ ④ ⑤	① ② ③ ④ ⑤	① ② ③ ④ ⑤	① ② ③ ④ ⑤
**Practice 3:** Provide health education curriculum or supplemental materials that include HIV, other STD, or pregnancy prevention information that are relevant to LGBTQ+ youth (e.g., curricula or materials that use inclusive language or terminology).	① ② ③ ④ ⑤	① ② ③ ④ ⑤	① ② ③ ④ ⑤	① ② ③ ④ ⑤	① ② ③ ④ ⑤
**Practice 4:** Encourage staff members to attend professional development on safe and supportive school environments for all students, regardless of sexual orientation, gender identity, or gender expression.	① ② ③ ④ ⑤	① ② ③ ④ ⑤	① ② ③ ④ ⑤	① ② ③ ④ ⑤	① ② ③ ④ ⑤
**Practice 5:** Facilitate access to providers not on school property who have experience providing social and behavioral health services to LGBTQ+ youth.	① ② ③ ④ ⑤	① ② ③ ④ ⑤	① ② ③ ④ ⑤	① ② ③ ④ ⑤	① ② ③ ④ ⑤
**Practice 6**: Facilitate access to providers not on school property who have experience providing sexual and reproductive health services (SRHS) to LGBTQ+ youth.	① ② ③ ④ ⑤	① ② ③ ④ ⑤	① ② ③ ④ ⑤	① ② ③ ④ ⑤	① ② ③ ④ ⑤

2. **Considering your rankings on the evaluation criteria above, decide as a team which TWO of the practices the Implementation Resource Team hopes to focus on this coming year. Identify action items in the table below.**

Practice	*Action Items for Year One*
